# Anaesthesia-Relevant Disease Manifestations and Perianaesthetic Complications in Patients with Mucolipidosis—A Retrospective Analysis of 44 Anaesthetic Cases in 12 Patients

**DOI:** 10.3390/jcm11133650

**Published:** 2022-06-24

**Authors:** Luise Sophie Ammer, Nicole Maria Muschol, René Santer, Annika Lang, Sandra Rafaela Breyer, Phillip Brenya Sasu, Martin Petzoldt, Thorsten Dohrmann

**Affiliations:** 1 International Center for Lysosomal Disorders (ICLD), Department of Paediatrics, University Medical Centre Hamburg-Eppendorf, 20246 Hamburg, Germany; muschol@uke.de (N.M.M.); r.santer.ext@uke.de (R.S.); annika.lang@stud.uke.uni-hamburg.de (A.L.); s.breyer@uke.de (S.R.B.); 2 Department of Anaesthesiology, University Medical Centre Hamburg-Eppendorf, 20246 Hamburg, Germany; p.sasu@uke.de (P.B.S.); m.petzoldt@uke.de (M.P.); t.dohrmann@uke.de (T.D.)

**Keywords:** mucolipidosis, ML, MLII, disease manifestations, symptoms, morbidity, anaesthesia, airway, perioperative complications, surgery

## Abstract

Mucolipidosis (ML) type II, intermediate, and III are lysosomal storage disorders with progressive multiorgan manifestations predisposing patients to a high risk of perioperative morbidity. The aims of the study were to systematically assess disease manifestations relevant to anaesthesia as well as anaesthesia-related complications. This retrospective study includes ML patients who underwent anaesthesia in two centres between 2008 and 2022. We reviewed patients’ demographics, medical history, disease manifestations, as well as procedure- and outcome-related data. A total of 12 patients (7 MLII, 2 ML intermediate, 3 MLIII) underwent 44 anaesthesia procedures (per patient: median 3, range 1–11). The median age was 3.3 years (range 0.1–19.1). At least one complication occurred in 27.3% of the anaesthesia procedures. The vast majority of complications (94%) occurred in children with MLII and ML intermediate. A predicted difficult airway was found in 100% and 80% of the MLII and ML intermediate patients, respectively. Accordingly, most complications (59%) occurred during the induction of anaesthesia. Altogether, respiratory complications were the most frequent (18%), followed by difficult airway management (14%). The risk for anaesthesia-related complications is alarmingly high in patients with ML, particularly in those with MLII and ML intermediate. Multidisciplinary risk–benefit analysis and thoughtful anaesthesia planning are crucial in these patients.

## 1. Introduction

Mucolipidosis type II (OMIM 252500; MLII), type III alpha/beta (OMIM 252600; MLIII alpha/beta), and type III gamma (OMIM 252605; MLIII gamma) are caused by pathogenic variants in the *GNPTAB*- and *GNPTG*-genes encoding for the N-acetylglucosamine (GlcNAc)-1-phosphotransferase [1]. This enzyme is involved in the intracellular trafficking of lysosomal enzymes by catalysing the first step of tagging a mannose-6-phosphate (M6P) recognition marker on newly synthesized lysosomal enzymes [2,3]. M6P-markers are required by most soluble lysosomal enzymes for their receptor-mediated transport over the Golgi network to the lysosomes. In MLII and III, an absent or reduced GlcNAc-1-phosphotransferase activity results in global mis-sorting of lysosomal enzymes and subsequent secretion into the extracellular compartment. Consecutively, partially degraded macromolecules (i.e., glycosaminoglycans, phospholipids, cholesterol) accumulate in the lysosomes impairing cellular function [4].

MLII is a rapidly progressing multi-systemic disease form with a fatal outcome, usually due to cardiorespiratory complications, within the first decade of life. Patients with MLIII commonly reach adulthood [5]. They present a later onset of symptoms, slower disease progression, and predominant skeletal symptoms [6,7]. ML patients who clinically cannot be attributed to either MLII or III are classified as ML intermediate. These patients are characterized by somatic findings similar to but slightly milder than MLII. The estimated incidence of MLII and III globally ranges from 0.22 to 2.70 per 100,000 live births [5]. No curative therapy is yet available [4].

Clinical features in ML overlap with those of patients with mucopolysaccharidosis (MPS), another more frequent lysosomal storage disorder. The overlapping progressive symptoms comprise craniofacial dysmorphism (i.e., flat face, shallow orbits, depressed nasal bridge, macroglossia), a short neck, respiratory insufficiency and upper airway obstruction, sleep apnoea, cardiac dysfunction, skeletal deformities (e.g., growth impairment, spine and chest deformity), and neurocognitive delay. Radiological features are subsumed as dysostosis multiplex [4,5,6,8,9]. For patients with MPS, these symptoms are known to be associated with a high risk of morbidity and mortality when undergoing general anaesthesia [10]. Direct laryngoscopy is especially challenging in these patient groups, causing high rates of failed airway management and cardiorespiratory events [11,12].

Thus far, anaesthesia-related complications have only been explored in single case reports [13,14] and small case series [12,15,16] of ML patients. Despite a suspected high incidence of complications in ML, a comprehensive analysis has not yet been performed. A clear understanding of the anaesthesia-relevant manifestations in different ML types and their effect on the anaesthetic risk is vital to improve clinical management and to avoid fatal outcomes related to difficult airway management. This study aims to ascertain the extent of anaesthesia-relevant disease manifestations as well as of perianaesthestic complications in patients with MLII, ML intermediate, and MLIII and to promote anaesthesia management recommendations.

## 2. Materials and Methods

### 2.1. Study Site and Patients

This cross-sectional study was conducted by retrospective chart review of patients of the International Centre for Lysosomal Disorders (ICLD) of the University Medical Center Hamburg-Eppendorf (UKE), Hamburg, Germany. Inclusion criteria were clinically and biochemically or molecular genetically confirmed diagnosis either of MLII, ML intermediate, or MLIII and at least one anaesthesia procedure (general anaesthesia; sedation; regional anaesthesia; monitored anaesthesia care) performed within the framework of the clinical routine between November 2008 and January 2022. The study cohort comprises cases from two centres, the UKE and the Children’s Hospital Altona (AKK), Hamburg, Germany.

### 2.2. Data Acquisition

The following information was extracted by systematic review of anaesthesia charts and analogue and electronic health records (Soarian Health Archive, Release 3.04 SP12, Siemens Healthcare, Erlangen, Germany): baseline characteristics, anaesthesia-relevant symptoms, and procedure- and outcome-related data. Baseline characteristics comprise the patients’ demographics and the medical history, including, amongst others, the underlying subtype (by clinical phenotype) and whether experimental hematopoietic stem cell transplantation (HSCT) or frequent respiratory infections was recorded.

As part of the standard of care, the patients underwent a multidisciplinary evaluation and diagnostic workup before elective anaesthesia. The following medical preconditions were considered anaesthesia-relevant and gathered by the time of anaesthesia (case): age, height, and weight; craniofacial dysmorphia; gingival hyperplasia; macroglossia; tonsil hyperplasia; short neck; cervical spinal stenosis; dens hypoplasia with atlantoaxial instability; spinal deformities; thorax deformities; sleep apnoea (as per polysomnography); obstructive lung disease; apparent dysphagia; cardiac pathologies; organomegaly (as per standard deviation of the index for body length); and growth retardation (as per body length and age- and gender-adapted Z-scores). Cardiac pathologies were described by the time of each anaesthesia procedure and categorized as recently published [10] as unremarkable, mild (plump valves; valvular defects Grades I/I–II, minor septum defects, atrioventricular block Grade I, left bundle branch block, arterial hypertension), moderate (valvular heart defects Grade II, discrete cardiac hypertrophy or dilative cardiomyopathy), or severe (valvular heart defects Grade III/IV, any manifestation requiring cardiac medication or intervention). In case of multiple anaesthesia procedures in one patient, the most pathologic finding determined the patient’s characteristics. As patients with ML intermediate present with severe and multi-systemic disease manifestations, we grouped patients with MLII and ML intermediate for descriptive analysis.

The following procedure-related data were collected for each study case: indication for anaesthesia; number of procedures during anaesthesia; duration; type of anaesthesia; primary airway approach; whether postoperative intensive care unit (ICU) care had been provided. 

The outcome parameters for perioperative complications were subdivided into four distinct entities: (1) difficult airway management, (2) respiratory, (3) cardiocirculatory, and (4) other intraoperative and postoperative complications documented until hospital discharge. Airway management and cardiorespiratory complications were defined as previously published [11].

### 2.3. Statistics

Data were collected in Microsoft Excel (Version 2011, Microsoft Corporation, Redmond, WA, USA) and analysed in R 4.0.3 (R core team, Vienna, Austria). Demographic data, disease manifestations, procedure-related factors, and outcome data were stratified by disease type and summarized as frequencies and percentages for categorical variables and as medians and ranges or means and standard deviations (SD) for continuous variables, as appropriate. The body metrics were collected at each patient contact, thus introducing a cluster structure. We used a mixed-effects linear model to correct for the repeated measurements within each patient using the lme4 package in R [17]. The use of natural cubic splines facilitated a non-linear relationship. Marginal means with corresponding 95% confidence intervals were calculated for the disease subtypes at different ages.

## 3. Results

### 3.1. Data Acquisition and Quality

We identified 22 patients with ML (13 MLII, 2 ML intermediate, 7 MLIII), of whom a subset of 12 (55%) had undergone at least 1 anaesthesia procedure in our centres during the study period of 13.3 years. The 12 enrolled patients underwent a total of 44 anaesthesia procedures. For all anaesthesia procedures, the protocols were available and readable. With a missing rate of 1%, the data quality is sufficient.

### 3.2. Patient Characteristics

#### 3.2.1. Patient Baseline Characteristics

The predominant phenotype in our study population was MLII (*n* = 7), followed by MLIII (*n* = 3) and, lastly, ML intermediate (*n* = 2). Two of the three MLIII patients were siblings. The median overall age at anaesthesia was 3.3 years (range 0.1–19.1). Patients with MLII and intermediate underwent intervention at a younger age than patients with MLIII. Among all, sex was even. One MLII patient underwent haematopoietic stem cell transplantation (HSCT) at nine months of age [18]. Altogether, the burden of anaesthesia-relevant symptoms was more pronounced in MLII and ML intermediate than in MLIII (Table 1).

#### 3.2.2. Growth

**Figure 1:**
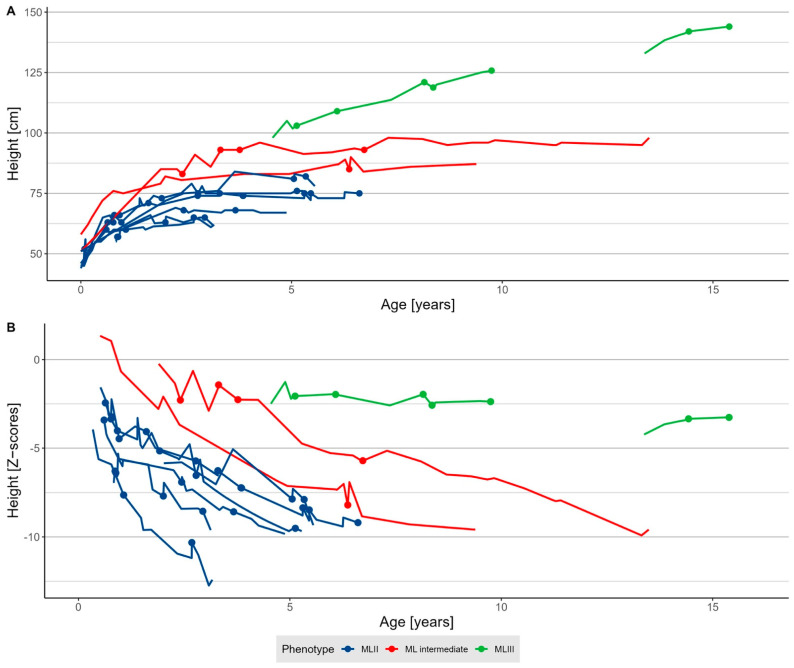
Growth in mucolipidosis. (**A**) Absolute height and (**B**) Z-scores [19] of patients with mucolipidosis type II (MLII, *n* = 7), intermediate (ML intermediate, *n* = 2), and III (MLIII, *n* = 3). Dots indicate the timepoints of anaesthesia procedures.

All of the MLII and intermediate patients presented severe growth failure. In MLII, vertical growth was only observed in infancy and in ML intermediate within the first three years of life (Figure 1 and Figure S1). Marginal means calculated from the mixed model showed a substantial height difference at the age of five years. Patients with MLII reached a mean, presumably maximum height of 73 cm (CI 69.4–76.6), and those with ML intermediate, a mean, presumably maximum height of 88.4 cm (CI 81.8–95). In contrast, the growth velocity of MLIII patients slowed down during childhood but did not cease. The mean height of MLIII patients was 104 cm (CI 97.5–111) at five years and 143 cm (CI 137.8–148.9) at 18 years of age.

#### 3.2.3. Upper Airway and Respiratory Tract Pathologies

Considering the severe growth retardation, patients with MLII and ML intermediate keep small thoraces and airways lifelong. Additional thorax deformities were documented in six MLII/intermediate patients, precisely an extremely narrow thorax (1/9; 11%) and pectus carinatum (5/9; 56%). Besides macroglossia, tonsil hyperplasia, and obstructive lung disease, the vast majority (8/9; 88%) of MLII/intermediate patients suffered from (obstructive or central) sleep apnoea, of which half (4/8) required non-invasive ventilation (NIV). NIV was initiated between one and six years of age.

#### 3.2.4. Cardiovascular Manifestations

All nine MLII/intermediate patients presented at least one cardiac pathology, 56% (5/9) of them even severe ones. Progressive valve insufficiency prevailed in all ML patients (9/12; 75%; mitral insufficiency *n* = 9, aortic insufficiency *n* = 6, tricuspid insufficiency *n* = 5). Septum defects were solely observed in MLII. One MLII patient suffering from a hemodynamically relevant atrial septal defect (ASD) type II was treated by catheter intervention at 4.9 years of age. A patent foramen ovale (PFO) and pulmonary artery stenosis were seen in the very young and heart failure rather in the longer-surviving children. The cardiac manifestations of the MLIII patients of this study were limited to mild to moderate valve insufficiency and sinus tachycardia.

#### 3.2.5. Gastrointestinal Manifestations

Dysphagia was manifested in 44% (4/9) of the MLII/intermediate patients, one MLII patient required enteral feeding, and 56% (5/9) of the MLII/intermediate patients had at least one relevant organomegaly.

#### 3.2.6. Spine Disease

Most of the MLII/intermediate children presented atlantoaxial instability (6/9; 67%) or stenosis of the craniocervical junction (7/9; 78%). One MLII patient even had cervical stenosis with myelopathy, and one ML intermediate patient had undergone cervical spine decompression surgery at the age of 2.8 years.

### 3.3. Characteristics of Anaesthesia Procedures

#### 3.3.1. Frequency of Anaesthesias and Technical Information

Anaesthesia care was provided for 12 patients with ML, accounting for 44 anaesthesia procedures (MLII *n* = 30; ML intermediate *n* = 5; MLIII *n* = 9). The majority of patients (83%) underwent more than one anaesthesia procedure (median 3, range 1–11). Anaesthesia procedures of MLII/intermediate patients (35/44; 80%) outnumbered those of MLIII patients (9/44; 20%). General anaesthesia was the preferred type of anaesthesia (33/44; 75%). In seven anaesthesia cases (7/44; 16%), procedural sedation was performed for diagnostics (primarily magnetic resonance imaging, MRI). Regional anaesthesia (2/44) was rarely applied. Tracheal intubation was carried out in 73% of the general anaesthesia cases (24/33) and a laryngeal mask airway in 27% (9/33). For most of the MLII/ML intermediate cases who were intubated, intubation was facilitated either by videolaryngoscopy (5/21; 24%) or fibreoptic intubation through a laryngeal mask (13/21; 62%). Therefore, in 86% (18/21) of the MLII/intermediate cases, an indirect intubation technique was used as the first-line airway approach. This was in contrast to patients with MLIII, for whom the laryngeal mask was the preferred primary airway approach for general anaesthesia (4/7; 57%).

The most frequently used anaesthesia regime was total intravenous anaesthesia with Propofol for induction and the maintenance of anaesthesia. Inhalative induction was only used in six cases (15%). Sufentanil and Remifentanil were the preferred analgesics for general anaesthesia. Detailed information about the anaesthesia procedures and drug combinations is presented in Table 2 and Supplementary Figure S2.

#### 3.3.2. Indication for Anaesthesia

**Figure 2:**
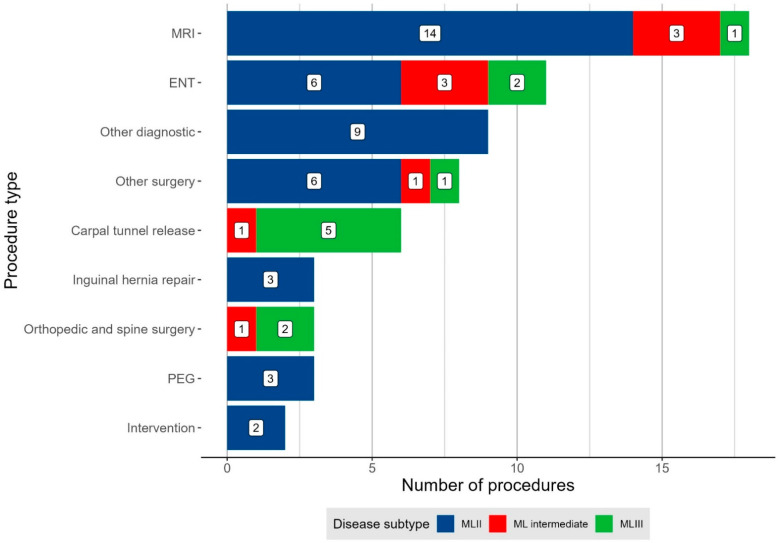
Indication for anaesthesia in all cases of patients with mucolipidosis. “Other diagnostic” sums up brainstem evoked response audiometry (BERA), ophthalmological, and neurophysiological assessments. “Other surgery” denotes all surgeries not represented in any other category and includes gingivectomy, dental, cardiac, and oncologic surgeries, and “intervention” indicates one lumbar puncture and one skin biopsy. Abbreviation: ENT, ear, nose, and throat surgery; MRI, magnetic resonance imaging; PEG, percutaneous endoscopic gastrostomy placement or exchange.

Two or more procedures were frequently combined within one anaesthesia. Anaesthesia was mainly performed for diagnostics (27/60; 45%; i.e., MRI *n* = 18; BERA *n* = 6; CT *n* = 1; ophthalmological and neurophysiological examinations *n* = 2). The most common surgical indications for anaesthesia were ear, nose, and throat (ENT) surgeries (11/60; 18%), carpal tunnel release (6/60; 10%), and hernia repair (3/60; 5%). Three quarters (9/12) of all ML patients underwent at least one ENT surgery. Carpal tunnel release was mainly performed in MLIII and hernia repair exclusively in MLII (Figure 2).

### 3.4. Outcome

Anaesthesia-related complications were common among ML patients. At least one anaesthesia-related complication occurred in 27.3% (12/44) of the anaesthesia procedures (Table 3). Altogether, 17 complications associated with anaesthesia occurred until discharge. Almost all complications (16/17; 94%) occurred in MLII/intermediate cases, in which the complication rate hence was increased compared to MLIII (31.4% vs. 11.1%).

More than half (10/17; 59%) of the complications arose during induction of anaesthesia, only 12% (2/17) during anaesthesia or extubation, and 29% (5/17) after anaesthesia. The most frequently experienced complications were respiratory complications (8/44; 18%) and difficult airway management (6/44; 14%). Respiratory complications, especially hypoxia, prevailed during the challenging airway management and extubation (5/8; 62.5% of the respiratory events). A predicted difficult airway was found in 100%, 80%, and 0% of the MLII, ML intermediate, and MLIII patients, respectively.

Success rates were 33% for direct laryngoscopy, 100% for videolaryngoscopy, and 92% for fibreoptic intubation through a laryngeal mask. Awake intubation was not performed. Facemask ventilation was difficult or impossible in five cases (14%). The laryngeal mask airway was difficult in only one case (4%). One child suffered from severe bradycardia with consecutive cardiac arrest due to hypoxia during difficult airway management. The placement of a laryngeal mask re-established sufficient oxygenation during critical events in all of the cases, at least temporarily. Subsequent rescue techniques comprised videolaryngoscopy and intubation via flexible optic with or without guidance via a laryngeal mask. Airway management was eventually successful in all cases so that no emergency tracheotomy or anaesthesia-related death occurred. None of the children experienced anaesthesia-related sequels. Individual case descriptions are presented in Table 4.

Of note, the data show a trend towards higher numbers of anaesthesia procedures over the study period of 13.3 years, while the number of complications remained relatively stable (Figure S3); hence, the risk of complications decreased over time.

## 4. Discussion

The present study is the largest published case series of patients with ML undergoing anaesthesia [11,12]. Furthermore, it is the first study to assess the full extent of anaesthesia-relevant multiorgan manifestations in this particularly rare disease. At least one complication arose during or after anaesthesia in more than a quarter of anaesthesia procedures. According to our data, difficult airway management and respiratory complications form the most alarming anaesthesia-relevant complications in patients with ML. Almost all complications occurred in patients with MLII and ML intermediate, even though indirect intubation techniques were favoured, as recommended in patients with skeletal dysplasia [20] and suggested in ML [11]. This correlates with the wide phenotypical spectrum between MLII and MLIII, with a much more pronounced anaesthesia-relevant disease burden in MLII and ML intermediate than in MLIII.

As pointed out recently [18], the natural history of ML is not yet described in detail. The present study specifies various disease manifestations relevant for anaesthesia planning. Although cardiorespiratory complications are widely known to be the leading cause of death in MLII [5], detailed data on the cardiac manifestations are sparse in the current literature [6,7]. Our study found frequent valve insufficiency and hypertrophic and dilated cardiomyopathy in the longer-surviving children, which is in agreement with previous reports [21,22]. In the sense of a continuum, similar cardiac manifestations may be present in MLIII as in MLII, but usually less severe or later in life. Interestingly, relevant cardiocirculatory complications have not been found in our case series.

The vast majority (89%) of the MLII and intermediate patients of this cohort suffered from sleep apnoea, which is in line with two case series [16,23] on sleep-disordered breathing in patients with MLII or intermediate, of whom 100% were documented to have obstructive sleep apnoea (OSA). The OSA turned out to be progressive with the eventual need for NIV in all children, which was inevitable with upper airway surgery (adenotonsillectomy). This can be explained by the multiple-level airway obstruction by the accumulation of undigested substrates in various body tissues leading to a hypertrophic tongue base, adenotonsillar hypertrophy, a thickened and retroflexed epiglottis, a thickened and anteriorly placed larynx, subglottic stenosis and a generally narrow and possibly curved trachea [12,15,24,25]. The craniofacial abnormalities, particularly the flat face and the depressed nasal bridge, also predispose patients to OSA, and cervical spine stenosis may precipitate central apnoea [26]. The high prevalence of OSA, as early as in infancy [23], fortifies the need for a systematic polysomnographic follow-up program in ML patients, as recommended for other lysosomal storage disorders [27]. Restrictive lung disease due to profound growth retardation, chest and spine deformities, and decreased thoracic elasticity may additionally contribute to respiratory insufficiency [8] and reduce the respiratory reserve during apnoeic episodes such as during airway management [28]. Pre-planned ICU monitoring may hence be beneficial for ML patients with severe disease forms. Of note, tonsil hyperplasia, sleep apnoea, and obstructive lung disease were also found in MLIII. All these symptoms lead to a high risk of perianaesthetic respiratory complications.

This is the first study to provide frequencies of different spinal pathologies in ML. Our study revealed a strikingly high percentage of stenosis of the craniocervical junction (78%) and atlantoaxial instability (67%) in patients with MLII and ML intermediate. Progressive cervical spinal cord compression with resulting respiratory failure and death has been described before [26]. However, not only patients with cervical spine pathologies need special attention, but also those with lower vertebral column abnormalities, which are also common in ML. The frequency of MLII children with thoracolumbar gibbus abnormality in this study (57%) roughly equals the percentage reported by Alegra et al. (50%) [29]. The literature contains a number of reports on children with MPS with neurological sequels after anaesthesia [10], such as an MPS IH patient with a 76° thoracic kyphosis and irreversible paraplegia after extremity surgery [30]. According to Farley et al., the spinal cord intramedullar pressure increases significantly in thoracic kyphosis exceeding 63° [31]. Cervical spinal stenosis and instability and thoracolumbar kyphosis may not be the predominant symptoms in MLIII. However, MLIII patients should also be handled with particular caution, considering that they may also present severe scoliosis and signs of spinal cord compression with possible loss of ambulation [6,7]. The high burden of spine disease highlights the importance of the careful positioning of all anaesthetized ML patients. Following this, we did not observe any neurological sequels in our patient cohort.

Due to their multiorgan morbidity, patients with ML frequently require anaesthesia for diagnostic or surgical interventions [7,11,12]. The patients in our study cohort had a median of three anaesthesias at our centres. We found a higher complication rate in MLII and intermediate than in MLIII (31.4% vs. 11.1%). Therefore, a more severe disease burden of patients with MLII and ML intermediate correlates with an increased risk for morbidity during and after anaesthesia. As indicated by Dohrmann et al. [11], the event rate in MLII (25%) is increased compared to MPSIII (9.1%) and certainly dramatically increased compared to the healthy paediatric population (0.14–5.2%) [32,33]. The majority of the anaesthesia procedures in case series of patients with MLII and III [12,15,16] published so far were described as challenging. However, several of these reported cases required emergency intubations because of acute respiratory distress associated with an infection [15,16]. One of the emergency intubations in a non-specialized hospital failed altogether with subsequent death [16]. Two other patients eventually required tracheotomy [15]. Considering the poor outcome of MLII patients intubated during respiratory distress, a restriction of elective anaesthesia to infection-free periods might be advisable. Furthermore, preoperative diagnostics allow for a multidisciplinary risk–benefit analysis of the planned intervention and improved counselling of the parents or patients. The event rate decreased over the study period of 13.3 years, and the anaesthesia management hence tendentially became safer. This underlines the importance of elective anaesthesias to be performed in tertiary hospitals with an ICU and a multidisciplinary team well-acquainted with a number of advanced airway management techniques. Nonetheless, the event rate should be interpreted with caution as the study population may have changed with improved disease detection and supportive clinical management.

The case number of this study remains too low to analyse perioperative management factors in order to deduce a standard of intraoperative care. We hence can only make suggestions based on the results of this study and a review of the literature [11,12,15,16]. ML patients have complex airways with predicted difficult airway management in 100% of MLII and 80% of ML intermediate patients. Indirect intubation methods such as videolaryngoscopy or fibreoptic intubation with guidance through a supraglottic conduit had the highest success rate in a previous study from our institution [11] and should be favoured for a first-line airway approach. With both methods, hyperextension of the head can be avoided [34]. However, our data indicate that, even with these prerequisites, a high risk for respiratory complications during airway management persists. A laryngeal mask airway can be used temporarily to re-establish stable ventilation during critical events [15]. Taking into account the severe growth retardation in patients with MLII and ML intermediate, anaesthesiologists should consider a smaller tube size than standard for the child’s age [12]. After all, the airway techniques should be chosen individually, depending on each patient’s complex manifestations and the distinct experience of the handling of anaesthesiologists and the institution.

This study has a few limitations. It is a retrospective study, so the documentation, including the anaesthesia-relevant complications, may have been incomplete. The small case number, especially of patients with MLIII, is based on limited patient availability: firstly, because ML is an extremely rare disease and, secondly, because preoperative risk-benefit considerations frequently trigger a decision against anaesthesia, and thus only patients with a mandatory indication for anaesthesia or an appealing risk–benefit ratio might have been selected for anaesthesia. Therefore, the true anaesthesia-relevant disease burden might even be higher. This centre’s experience may have caused a selection bias, so our results should only be generalized with caution.

## 5. Conclusions

Featuring 12 ML patients who underwent anaesthesia on 44 occasions, the present study is currently the largest study published on anaesthetic risk in ML. Moreover, it is the first to give full particulars on anaesthesia-relevant disease manifestations among the different ML types. At least one complication occurred in 27.3% of the anaesthesia procedures, almost all (94%) in patients with MLII or ML intermediate. This finding corresponds with a much more pronounced anaesthesia-relevant disease burden in MLII and ML intermediate than in MLIII. Most complications (59%) occurred during critical airway management. Hence, we suggest indirect intubation techniques (i.e., videolaryngoscopy and fibreoptic intubation through a supraglottic airway) to be routinely used for the a priori airway approach in these children. This study highlights the necessity of individualized preoperative risk–benefit analysis, shared and multidisciplinary decision-making, and thoughtful anaesthesia planning in patients with ML.

## Figures and Tables

**Table 1:** Characteristics and anaesthesia-relevant symptoms of patients with mucolipidosis.

Characteristic	Overall, *N* = 12	MLII, *N* = 7	MLII/III, *N* = 2	MLIII, *N* = 3
Demographics				
Male sex ^2^	6 (50)	4 (57)	1 (50)	1 (33)
Minimum age at anaesthesia ^1^	1.7 (0.1–19.0)	0.9 (0.1–5.1)	4.4 (2.4–6.4)	14.4 (5.1–19.0)
Maximum age at anaesthesia ^1^	5.9 (0.8–19.1)	3.7 (0.8–6.6)	6.5 (6.4–6.7)	15.4 (8.4–19.0)
Medical history				
Number of anaesthesias ^1^	4 (1–11)	4 (2–11)	2 (1–4)	3 (1–5)
HSCT ^2^	1 (8)	1 (14)	-	-
History of recurrent infections ^2^	2 (17)	2 (29)	-	1 (33)
Upper airway and respiratory tract pathology				
Craniofacial dysmorphia ^2^	10 (83)	7 (100)	2 (100)	1 (33)
Gingival hyperplasia ^2^	9 (75)	7 (100)	2 (100)	-
Macroglossia ^2^	6 (50)	5 (71)	1 (50)	-
Tonsil hyperplasia ^2^	6 (50)	2 (29)	2 (100)	2 (67)
Sleep apnoea ^2^				
No	3 (25)	1 (14)	-	2 (67)
Yes	5 (42)	2 (29)	2 (100)	1 (33)
NIV-treated	4 (33)	4 (57)	-	-
Obstructive lung disease ^2^				
Yes	5 (42)	4 (57)	-	1 (33)
Antiobstructive medication	2 (17)	1 (14)	1 (50)	-
Thorax deformities ^2^				
Pectus carinatum	5 (42)	4 (57)	1 (50)	-
Narrow thorax	1 (8.3)	1 (14)		-
Cardiovascular manifestations				
Severity of cardiac pathologies ^2^				
Mild	3 (25)	1 (14)	1 (50)	1 (33)
Moderate	3 (25)	2 (29)	-	1 (33)
Severe	5 (42)	4 (57)	1 (50)	-
Cardiac pathology ^2^				
Valve insufficiency	9 (75)	5 (71)	2 (100)	2 (67)
ASD type II	5 (42)	5 (71)	-	-
LVH	4 (33)	4 (57)	-	-
PFO	2 (17)	2 (29)	-	-
PA stenosis	2 (17)	2 (29)	-	-
Heart failure	2 (17)	1 (14)	1(50)	
LV dilatation	1 (8)	1 (14)	-	-
Hypertension	1 (8)	1 (14)	-	-
Tachycardia	1 (8)	-	-	1 (33)
Gastrointestinal manifestations				
Organomegaly ^2^				
Hepatomegaly	4 (33)	4 (57)	-	-
Hepatosplenomegaly	1 (8)	1 (14)	-	-
Dysphagia ^2^				
Yes	4 (33)	3 (43)	1 (50)	-
Gastric tube	1 (8)	1 (14)	-	-
Spine disease				
Short neck ^2^	12 (100)	7 (100)	2 (100)	3 (100)
Cervical spinal stenosis^2^				
Stenosis	5 (42)	4 (57)	1 (50)	-
Stenosis + myelopathy	1 (8)	1 (14)	-	-
State after surgical decompression	1 (8)	-	1 (50)	-
Cervical spinal instability ^2^	6 (50)	4 (57)	2 (100)	-
Spinal deformities ^2^				
Thoracolumbar kyphosis	5 (42)	4 (57)	1 (50)	-
Lumbar hyperlordosis	1 (8)	-	-	1 (33)
Kyphoscoliosis	1 (8)	-	1(50)	-

Abbreviations: ASD type II, atrial septal defect type II; NIV, non-invasive ventilation; LVH, left ventricular hypertrophy; PA stenosis, pulmonary artery stenosis; PFO, patent foramen ovale. ^1^ median (range), ^2^ *n* (%).

**Table 2:** Characteristics of anaesthesia procedures.

Characteristic	Overall, *N* = 44	MLII, *N* = 30	MLII/III, *N* = 5	MLIII, *N* = 9
Cases				
Age (years) ^1^	3.3 (0.1; 19.1)	2.2 (0.1; 6.6)	3.8 (2.4; 6.7)	9.7 (5.1; 19.1)
Weight (Z-score) ^1^	−3.6 (−13.1; 0.3)	−4.8 (−13.1; −0.6)	−1.7 (−4.6; −0.7)	−0.8 (−2.5; −0.3)
Height (Z-score) ^1^	−5.7 (−10.3; −1.4)	−6.6 (−10.3; −2.4)	−2.3 (−8.2; −1.4)	−2.37 (−3.3; −2.0)
Present respiratory infection ^2^	6 (14)	4 (13)	2 (40)	-
ASA score ^2^				
2	8 (18)	-	1 (20)	7 (78)
3	32 (73)	27 (90)	3 (60)	2 (22)
4	4 (9)	3 (10)	1 (20)	0 (0)
Procedural information				
Number of procedures during anaesthesia ^2^	1.0 (1.0; 6.0)	1.0 (1.0; 6.0)	2.0 (1.0; 3.0)	1.0 (1.0; 2.0)
Duration (minutes) ^1^	120 (55; 405)	122 (55; 270)	270 (90; 405)	105 (60; 240)
Postoperative ICU care ^2^	24 (56)	19 (63)	5 (100)	-
Technical information				
Type of anaesthesia ^2^				
Standby	2 (5)	1 (3)	-	1 (11)
Sedation	7 (16)	7 (23)	-	-
Regional only	2 (5)	1 (3)	-	1 (11)
General anaesthesia	33 (75)	21 (70)	5 (100)	7 (78)
Total intravenous anaesthesia	29 (66)	17(57)	5 (100)	7 (78)
Balanced anaesthesia	4 (9)	4 (13)	-	-
Induction of anaesthesia				
Intravenous (Propofol)	34 (85)	25 (89)	3 (60)	6 (86)
Inhalative (Sevoflurane)	6 (15)	3 (11)	2 (40)	1 (14)
Primary airway approach ^2^				
No airway	11 (25)	9 (30)	-	2 (22)
Laryngeal mask	9 (20)	5 (17)	-	4 (44)
Tracheal intubation	24 (55)	16 (53)	5 (100)	3 (33)
Direct laryngoscopy	3 (7)	2 (7)	1 (20)	-
Videolaryngoscopy	8 (18)	5 (17)	-	3 (33)
FOI-SGA	13 (30)	9 (30)	4 (80)	-

Abbreviations: ASA score, American Society of Anesthesiology score; FOI-SGA, fiberoptic through a supraglottic airway; ICU, intensive care unit; TIVA, total intravenous anaesthesia. Z-Score derived from Kromeyer-Hauschild et al. [19]. ^1^ median (range), ^2^ *n* (%).

**Table 3:** Anaesthesia-related complications subdivided by ML types.

Characteristic, *n (%)*	Overall, *N* = 44	MLII, *N* = 30	MLII/III, *N* = 5	MLIII, *N* = 9
Anaesthesias with at least one complication	12 (27)	8 (27)	3 (60)	1 (11)
Difficult airway management	6 (14)	5 (17)	1 (20)	-
Difficult facemask ventilation	5 (14)	4 (18)	1 (20)	-
Difficult laryngeal mask airway	1 (4)	1 (6.2)	-	-
Difficult tracheal intubation	6 (23)	5 (29)	1 (20)	-
Respiratory complications	8 (18)	6 (20)	2 (40)	-
Cardiocirculatory complications	-	-	-	-
Other complications	3 (7)	1 (3)	1 (20)	1 (11)

**Table 4:** Details of perianaesthetic complications in patients with ML.

Pat.	Sex	Subtype	Year	Age	Procedure	Airway Management	Anaesthesia Procedure	Event Categories	Detailed Descriptions
1	Female	MLII	2020	3 years	PEG exchange	VL → FOI-LM	TIVA: Propofol, Remifentanil	DiffAir, RESP	Videolaryngoscopy: failed, C/L 4 view; FOI-LM: passage of 4.0 tube failed, successful placement of 3.5 uncuffed tube; tube exchange because of significant air leak (3.5 cuffed tube via exchange catheter); hypoxemia with SpO_2_ 75%; unplanned ICU admission
1	Female	MLII	2019	2 years	MRI, BERA	No airway	Sedation: Propofol, Esketamine	RESP	Fever, bronchopneumonia treated with i.v. antibiotics
1	Female	MLII	2017	10 months	MRI, lumbar puncture	No airway → LM → VL → FOI-LM	Sedation: Propofol, Esketamine	DiffAir, RESP, other	Sedation: failed due to insufficient spontaneous breathing; difficult face mask ventilation; tracheal intubation: failed (conventional and videolaryngoscopy VL: C/L 3; hypoxemia and consecutive severe bradycardia requiring CPR; LM (rescue manoeuvre) facilitated oxygenation, ROSC, massive hypercapnia; FOI-LM: finally successful; postoperative respiratory insufficiency with prolonged ventilation on ICU; sepsis on ICU
2	Male	MLII/III	2008	6 years	ENT surgery	FOI-LM	TIVA: Propofol, Sufentanil	RESP	Postoperative tube dislocation into the main bronchus with atelectasis
3	Male	MLIII	2020	8 years	hip osteotomy	VL	Sevoflurane, TIVA: Propofol, Remifentanil	Other	Postoperative fever
4	Male	MLII	2021	2 years	ENT surgery	FOI-LM → VL → FOI	TIVA: Propofol, Remifentanil	DiffAir, RESP	Impossible face mask ventilation; FOI-LM: failed due to secretion and unfavourable angle; VL failed (C/L 3). Finally, tracheal intubation was secured by oral FOI. Hypoxemia with minimal SpO_2_ 58%
5	Male	MLII	2017	1 year	MRI	FOI-LM	TIVA: Propofol, Remifentanil	RESP	Hypoxemia with minimal SpO_2_ of 70% during intubation
5	Male	MLII	2016	11 months	ENT surgery	DL	TIVA: Propofol, Sufentanil	DiffAir	Impossible face mask ventilation, LM; tracheal intubation via direct laryngoscopy
6	Female	MLII	2014	7 months	Quinton catheter implantation	DL → other	Balanced: Propofol, Sufentanil, Sevoflurane	DiffAir	Difficult intubation; conventional laryngoscopy C/L 3; successful intubation with a rigid bronchoscope
7	Female	MLII/III	2015	6 years	ENT surgery, MRI	FOI-LM	TIVA: Propofol, Sufentanil	Other	Postoperative fever
7	Female	MLII/III	2012	3 years	Atlanto-occipital decompression	DL → VL → other	Sevoflurane TIVA: Propofol, Sufentanil	DiffAir, RESP	Difficult mask ventilation; VL: C/L 3; intubation with a McCoy blade and rigid bronchoscope, hypoxemia
8	Male	MLII	2021	5 years	ENT surgery, MRI, other diagnostics	VL	TIVA: Propofol, Remifentanil	RESP	Postextubation airway obstruction with severe hypoxemia (SpO_2_ 8%)

Abbreviations: BERA, brainstem electric response audiometry; C/L, Cormack/Lehane; CPR, cardiopulmonary resuscitation; DiffAir, difficult airway management; DL, direct laryngoscopy; ENT, ear, nose, and throat; FOI, fibreoptic intubation; LM, laryngeal mask; FOI-LM, fibreoptic intubation guided by laryngeal mask; GA, general anaesthesia; ICU, intensive care unit; MRI, magnetic resonance imaging; PEG, percutaneous endoscopic gastrostomy; RESP, respiratory event; ROSC, return of spontaneous circulation; VL, videolaryngoscopy; TIVA, total intravenous anaesthesia.

## Data Availability

The data that support the findings of this study are available from the corresponding author upon reasonable request. The data are not publicly available to ensure that the privacy of the study patients with rare diseases is not compromised.

## Supplementary Materials

The following supporting information can be downloaded at: https://www.mdpi.com/article/10.3390/jcm11133650/s1, Figure S1: Estimated marginal means of the height of patients with mucolipidosis; Figure S2: Frequency of drug combinations used during general anaesthesia and sedation; Figure S3: Centre experience.

## References

[1] Tiede S., Storch S., Lubke T., Henrissat B., Bargal R., Raas-Rothschild A., Braulke T. (2005). Mucolipidosis II is caused by mutations in GNPTA encoding the alpha/beta GlcNAc-1-phosphotransferase. Nat. Med..

[2] Braulke T., Bonifacino J.S. (2009). Sorting of lysosomal proteins. Biochim. Biophys. Acta.

[3] Kollmann K., Pohl S., Marschner K., Encarnacao M., Sakwa I., Tiede S., Poorthuis B.J., Lübke T., Müller-Loennies S., Storch S. (2010). Mannose phosphorylation in health and disease. Eur. J. Cell Biol..

[4] Khan S.A., Tomatsu S.C. (2020). Mucolipidoses Overview: Past, Present, and Future. Int. J. Mol. Sci..

[5] Dogterom E.J., Wagenmakers M., Wilke M., Demirdas S., Muschol N.M., Pohl S., van der Meijden J.C., Rizopoulos D., van der Ploeg A.T., Oussoren E. (2021). Mucolipidosis type II and type III: A systematic review of 843 published cases. Genet. Med. Off. J. Am. Coll. Med. Genet..

[6] Cathey S.S., Leroy J.G., Wood T., Eaves K., Simensen R.J., Kudo M., Stevenson R.E., Friez M.J. (2010). Phenotype and genotype in mucolipidoses II and III alpha/beta: A study of 61 probands. J. Med. Genet..

[7] Oussoren E., van Eerd D., Murphy E., Lachmann R., van der Meijden J.C., Hoefsloot L.H., Verdijk R., Ruijter G.J.G., Maas M., Hollak C.E.M. (2018). Mucolipidosis type III, a series of adult patients. J. Inherit. Metab. Dis..

[8] Velho R.V., Harms F.L., Danyukova T., Ludwig N.F., Friez M.J., Cathey S.S., Filocamo M., Tappino B., Güneş N., Tüysüz B. (2019). The lysosomal storage disorders mucolipidosis type II, type III alpha/beta, and type III gamma: Update on GNPTAB and GNPTG mutations. Hum. Mutat..

[9] Ammer L.S., Oussoren E., Muschol N.M., Pohl S., Rubio-Gozalbo M.E., Santer R., Stuecker R., Vettorazzi E., Breyer S.R. (2020). Hip Morphology in Mucolipidosis Type II. J. Clin. Med..

[10] Ammer L.S., Dohrmann T., Muschol N.M., Lang A., Breyer S.R., Ozga A.K., Petzoldt M. (2021). Disease Manifestations in Mucopolysaccharidoses and Their Impact on Anaesthesia-Related Complications—A Retrospective Analysis of 99 Patients. J. Clin. Med..

[11] Dohrmann T., Muschol N.M., Sehner S., Punke M.A., Haas S.A., Roeher K., Breyer S., Koehn A.F., Ullrich K., Zöllner C. (2020). Airway management and perioperative adverse events in children with mucopolysaccharidoses and mucolipidoses: A retrospective cohort study. Paediatr. Anaesth..

[12] Scott-Warren V.L., Walker R. (2021). Perioperative management of patients with Mucolipidosis II and III: Lessons from a case series. Paediatr. Anaesth..

[13] Mahfouz A.K., George G. (2011). Anesthesia for gingivectomy and dental extractions in a child with I-cell disease—A case report. Middle East J. Anaesthesiol..

[14] Mahfouz A.K., George G., Al-Bahlani S.S., Al Nabhani M.Z. (2010). Difficult intubation management in a child with I-cell disease. Saudi J. Anaesth..

[15] Mallen J., Highstein M., Smith L., Cheng J. (2015). Airway management considerations in children with I-cell disease. Int. J. Pediatric Otorhinolaryngol..

[16] Edmiston R., Wilkinson S., Jones S., Tylee K., Broomfield A., Bruce I.A. (2019). I-Cell Disease (Mucolipidosis II): A Case Series from a Tertiary Paediatric Centre Reviewing the Airway and Respiratory Consequences of the Disease. JIMD Rep..

[17] Bates D., Maechler M., Bolker B., Walker S. (2015). Fitting Linear Mixed-Effects Models Using lme4. J. Stat. Softw..

[18] Ammer L.S., Pohl S., Breyer S.R., Aries C., Denecke J., Perez A., Petzoldt M., Schrum J., Müller I., Muschol N.M. (2021). Is hematopoietic stem cell transplantation a therapeutic option for mucolipidosis type II?. Mol. Genet. Metab. Rep..

[19] Kromeyer-Hauschild K., Wabitsch M., Kunze D., Geller F., Geiß H.C., Hesse V., Von Hippel A., Jaeger U., Johnsen D., Korte W. (2001). Perzentile für den Body-mass-Index für das Kindes- und Jugendalter unter Heranziehung verschiedener deutscher Stichproben. Mon. Kinderheilkd..

[20] White K.K., Bompadre V., Goldberg M.J., Bober M.B., Cho T.J., Hoover-Fong J.E., Irving M., Mackenzie W.G., Kamps S.E., Raggio C. (2017). Best practices in peri-operative management of patients with skeletal dysplasias. Am. J. Med. Genet. Part A.

[21] Dangel J.H. (1998). Cardiovascular changes in children with mucopolysaccharide storage diseases and related disorders—Clinical and echocardiographic findings in 64 patients. Eur. J. Pediatrics.

[22] Carboni E., Sestito S., Lucente M., Morrone A., Zampini L., Chimenz R., Ceravolo M.D., De Sarro R., Ceravolo G., Calabrò M.P. (2020). Dilated cardiomyopathy in mucolipidosis type 2. J. Biol. Regul. Homeost. Agents.

[23] Tabone L., Caillaud C., Amaddeo A., Khirani S., Michot C., Couloigner V., Brassier A., Cormier-Daire V., Baujat G., Fauroux B. (2019). Sleep-disordered breathing in children with mucolipidosis. Am. J. Med. Genet. Part A.

[24] Peters M.E., Arya S., Langer L.O., Gilbert E.F., Carlson R., Adkins W. (1985). Narrow trachea in mucopolysaccharidoses. Pediatr. Radiol..

[25] Poore T.S., Prager J., Weinman J.P., Larson A., Houin P. (2020). Tracheal and lower airway changes in a patient with mucolipidosis type II. Pediatr. Pulmonol..

[26] Nakaoka S., Kondo H., Matsuoka K., Shibuya T., Otomo T., Hamada Y., Sakamoto K., Ozono K., Sakai N. (2021). Mucolipidosis Ⅱ and III with neurological symptoms due to spinal cord compression. Brain Dev..

[27] Scarpa M., Almássy Z., Beck M., Bodamer O., Bruce I.A., De Meirleir L., Guffon N., Guillén-Navarro E., Hensman P., Jones S. (2011). Mucopolysaccharidosis type II: European recommendations for the diagnosis and multidisciplinary management of a rare disease. Orphanet J. Rare Dis..

[28] Wooten W.I., Muhlebach M.S., Muenzer J., Loughlin C.E., Vaughn B.V. (2016). Progression of Polysomnographic Abnormalities in Mucolipidosis II (I-Cell Disease). J. Clin. Sleep Med..

[29] Alegra T., Sperb-Ludwig F., Guarany N.R., Ribeiro E.M., Lourenco C.M., Kim C.A., Valadares E.R., Galera M.F., Acosta A.X., Horovitz D.D.G. (2019). Clinical Characterization of Mucolipidoses II and III: A Multicenter Study. J. Pediatric Genet..

[30] Pruszczynski B., Mackenzie W.G., Rogers K., White K.K. (2015). Spinal Cord Injury After Extremity Surgery in Children with Thoracic Kyphosis. Clin. Orthop. Relat. Res..

[31] Farley C.W., Curt B.A., Pettigrew D.B., Holtz J.R., Dollin N., Kuntz C. (2012). Spinal cord intramedullary pressure in thoracic kyphotic deformity: A cadaveric study. Spine.

[32] Habre W., Disma N., Virag K., Becke K., Hansen T.G., Jöhr M., Leva B., Morton N.S., Vermeulen P.M., Zielinska M. (2017). Incidence of severe critical events in paediatric anaesthesia (APRICOT): A prospective multicentre observational study in 261 hospitals in Europe. Lancet Respir. Med..

[33] Kurth C.D., Tyler D., Heitmiller E., Tosone S.R., Martin L., Deshpande J.K. (2014). National pediatric anesthesia safety quality improvement program in the United States. Anesth. Analg..

[34] Lee J.J., Lim B.G., Lee M.K., Kong M.H., Kim K.J., Lee J.Y. (2012). Fiberoptic intubation through a laryngeal mask airway as a management of difficult airwary due to the fusion of the entire cervical spine—A report of two cases. Korean J. Anesthesiol..

