# Supplementary material for: Anaesthesia-Relevant Disease Manifestations and Perianaesthetic Complications in Patients with Mucolipidosis—A Retrospective Analysis of 44 Anaesthetic Cases in 12 Patients
Source: J Clin Med. 2022 Jun 24;11(13):3650. doi: 10.3390/jcm11133650 (PMC9267794; doi:10.3390/jcm11133650)
Supplement: Supplementary file 1 [file jcm-11-03650-s001.zip › jcm-1753841-supplementary.pdf]

Journal of
Clinical Medicine

MDPI

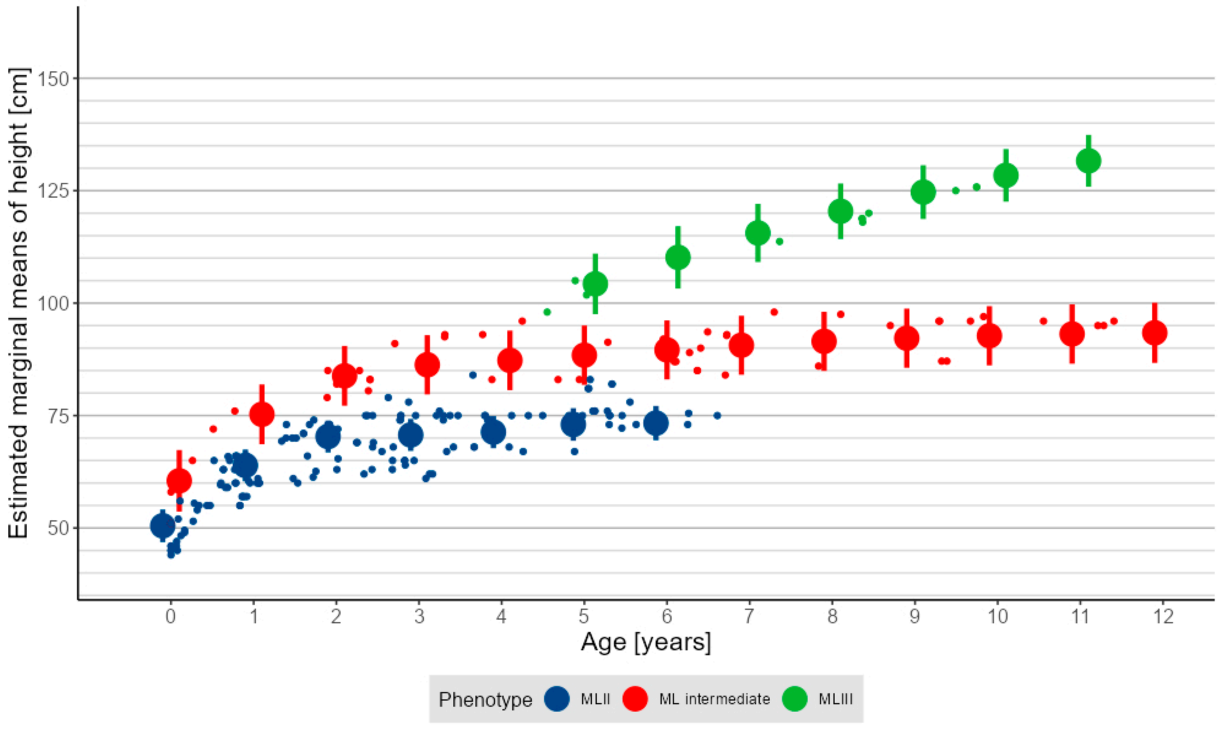

Figure S1. Estimated marginal means of the height of patients with mucolipidosis (MLII n=7, ML intermediate n=2, MLIII n=3).

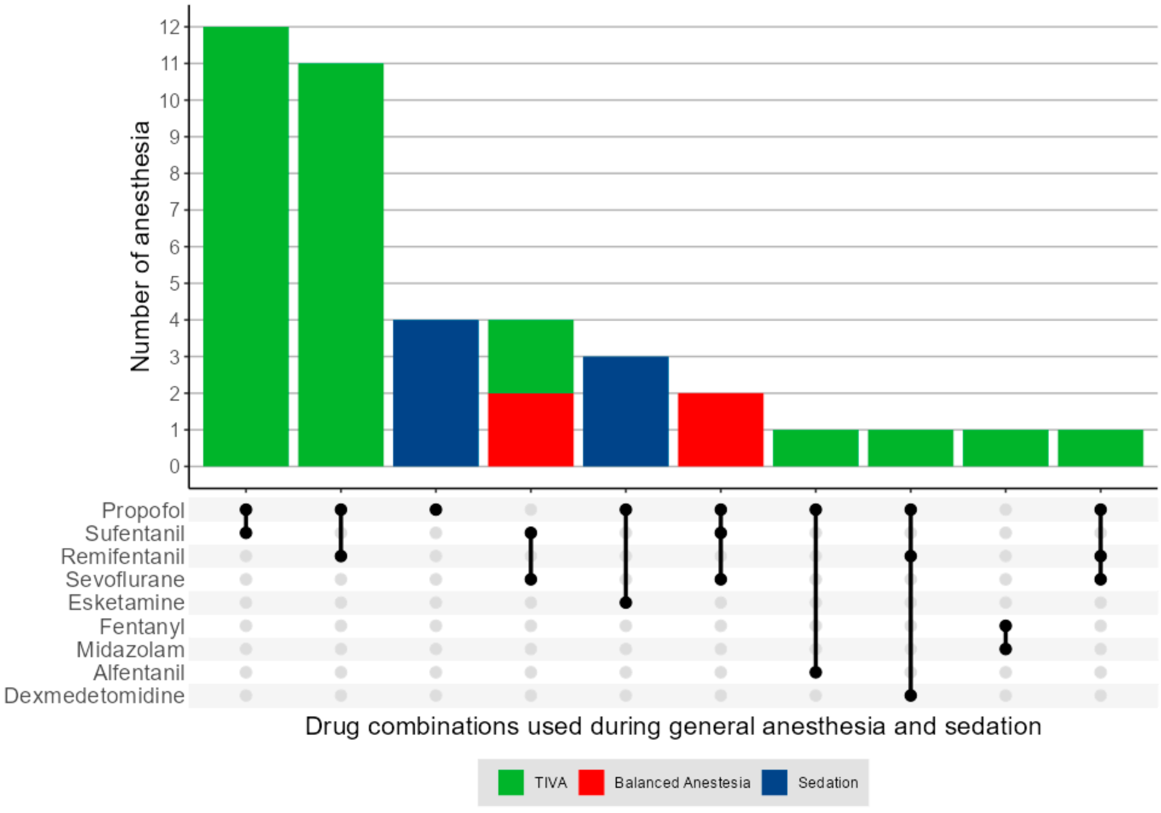

Figure S2. Frequency of drug combinations used during general anesthesia and sedation. TIVA, total intravenous anesthesia.

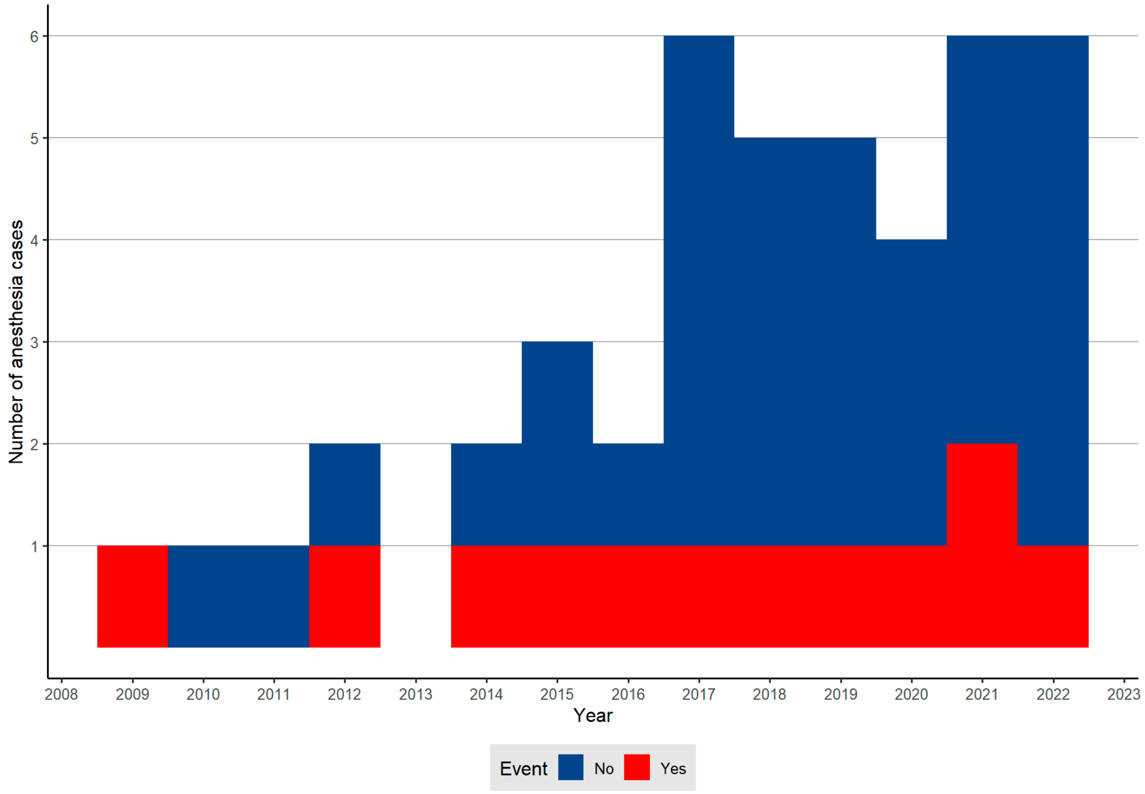

Figure S3. Center experience. Cases numbers of patients with mucolipidosis undergoing anaesthesia with and without complications during the study.
